# Artificial Intelligence-Based Classification and Segmentation of Bladder Cancer in Cystoscope Images

**DOI:** 10.3390/cancers17010057

**Published:** 2024-12-28

**Authors:** Won Ku Hwang, Seon Beom Jo, Da Eun Han, Sun Tae Ahn, Mi Mi Oh, Hong Seok Park, Du Geon Moon, Insung Choi, Zepa Yang, Jong Wook Kim

**Affiliations:** 1 Department of Urology, Korea University Guro Hospital, Korea University College of Medicine, Seoul 08308, Republic of Korea; 94525wg@naver.com (W.K.H.); rhfughfkddl@naver.com (S.B.J.); asturology@gmail.com (S.T.A.); mamah@hanmail.net (M.M.O.); dr4you@korea.ac.kr (H.S.P.); dgmoon@korea.ac.kr (D.G.M.); 2 Department of Radiology, Korea University Guro Hospital, Seoul 08308, Republic of Koreayangzepa@korea.ac.kr (Z.Y.)

**Keywords:** bladder cancer, cystoscopy, artificial intelligence, deep learning

## Abstract

Background/Objectives: Cystoscopy is necessary for diagnosing bladder cancer, but it has limitations in identifying ambiguous lesions, such as carcinoma in situ (CIS), which leads to a high recurrence rate of bladder cancer. With the significant advancements in deep learning in the medical field, several studies have explored its application in cystoscopy. This study aimed to utilize the VGG19 and Deeplab v3+ deep learning models to classify and segment cystoscope images, respectively. Methods: We classified cystoscope images obtained from 772 patients based on morphology (normal, papillary, flat, mixed) and biopsy results (normal, Ta, T1, T2, CIS, etc.). Experienced urologists annotated and labeled the lesion areas and image categories. The classification model for bladder cancer lesion, annotated with pathological results, was developed using VGG19 with an additional fully connected layer, utilizing sparse categorical cross-entropy as the loss function. The Deeplab v3+ model was used for segmenting each morphological type of bladder cancer in the cystoscope images, employing the dice coefficient loss function. The classification model was evaluated using validation accuracy and correlation with biopsy results, while the segmentation model was assessed using the Intersection over Union (IoU) combined with binary accuracy. Results: The dataset was split into training and validation sets with a 4:1 ratio. The VGG19 classification model achieved an accuracy score of 0.912. The Deeplab v3+ segmentation model achieved an IoU of 0.833 and a binary accuracy of 0.951. Visual analysis revealed a high similarity between the lesions identified by Deeplab v3+ and those labeled by experts. Conclusions: In this study, we applied two deep learning models using well-annotated datasets of cystoscopic images. Both VGG19 and Deeplab v3+ demonstrated high performance in classification and segmentation, respectively. These models can serve as valuable tools for bladder cancer research and may aid in the diagnosis of bladder cancer.

## Simple Summary

While the cystoscope is crucial for the diagnosis and treatment of bladder cancer, it shows limitations in distinguishing ambiguous lesions, such as small or flat lesions. To overcome these limitations, various studies have utilized deep learning in cystoscope image evaluation. In this study, which performed classification and segmentation of bladder lesions in cystoscope images using a deep learning model, the VGG19 and Deeplab v3+ models showed high accuracy in classification and segmentation, respectively.

## 1. Introduction

Bladder cancer was the ninth most commonly diagnosed cancer worldwide in 2022 [1]. According to the American Cancer Society’s 2024 estimates, approximately 83,190 new cases of bladder cancer are expected in the United States alone, accounting for about 4.2% of all new cancer cases in the country. The incidence of bladder cancer increases dramatically with age, particularly after 50–54 years, and shows a notable gender disparity with men having about three times higher incidence rates than women.

The identification of lesions through cystoscopy and biopsy is essential for diagnosing bladder cancer. Cystoscopy allows direct observation of the urethra and bladder interior, enabling the detection of most papillary and sessile bladder tumors. However, cystoscopy is not 100% accurate and has several limitations. For instance, studies have reported cystoscopy to have a sensitivity of 81% and specificity of 73%, indicating a significant risk of missing or misdiagnosing bladder tumors [2].

While mortality from bladder cancer is relatively low, the major concern lies in recurrence and progression. The high recurrence rate of early cancer is often attributed to the limitations of cystoscopy, which makes it difficult to distinguish ambiguous lesions such as carcinoma in situ (CIS) and to accurately delineate the boundaries of the tumors. Moreover, it is well known that the accuracy of bladder cancer diagnosis during cystoscopy varies depending on the physician’s level of expertise. A study based on SEER data in the United States demonstrated that treatment outcomes for metastatic urothelial carcinoma of the urinary bladder (mUCUB) differ by region [3]. These highlight the critical need for developing standardized diagnostic and treatment protocols to ensure equitable and consistent care.

To overcome these limitations, various research and technological developments are underway. Enhanced imaging techniques such as narrow band imaging (NBI) and photodynamic diagnosis (PDD) are contributing to improved tumor detection rates. Integrated technological approaches, such as combining micro-enhancement techniques with fluorescence imaging technologies, are being actively explored to improve diagnostic accuracy. Also, the development of non-invasive bladder cancer diagnostic methods using urinary biomarkers is gaining significant attention. Additionally, research utilizing artificial intelligence (AI) and machine learning (ML) technologies for real-time tumor detection, histological classification, and risk assessment is actively progressing.

Deep learning has made significant advancements and has found widespread application in diagnosing and treating various diseases. Many deep learning models, including convolutional neural networks (CNNs), are being used in medical imaging, pathology analysis, and other fields. CNNs have shown powerful performance in image classification and segmentation, making them one of the most pivotal deep learning models [4,5]. In the case of bladder cancer, several studies have applied CNNs to urine cytology analysis [6], CT image segmentation [7,8], and cystoscope image evaluations.

Previous studies have demonstrated the performance of deep learning models in segmenting bladder cancer on cystoscope images [9,10,11]; Ikeda et al. achieved a sensitivity of 89.7% and specificity of 94.0% using a GoogLeNet-based CNN model, while Shkolyar et al.’s CystoNet demonstrated a sensitivity of 90.9% and specificity of 98.6%. Guo et al. further utilized a U-Net algorithm to achieve 98% accuracy in detecting bladder cancer lesions from 3500 images. However, no studies have yet conducted the classification of bladder lesion images using deep learning based on the pathological results of each lesion. In our study, we seek to bridge this gap by conducting deep learning-based classification of bladder lesions using texture and biopsy results from cystoscope images. Additionally, we applied Deeplab v3+ for the segmentation of bladder cancer lesions in cystoscopic images.

In the main text, we will describe the deep learning models we employed, namely VGG19 and Deeplab v3+, and explain the results of classification and segmentation using these models. In the discussion section, we will review current ongoing research related to cystoscopy and address the advantages and limitations of the deep learning models we utilized. Finally, we will discuss the significance and limitations of our study, propose potential improvements, and outline future research directions.

## 2. Materials and Methods

### 2.1. Study Population

We conducted a study involving patients who underwent cystoscopy either for follow-up after a previous bladder cancer diagnosis or due to suspected bladder cancer indicated by hematuria, ultrasound findings of bladder tumors, or other symptoms. Among the patients who underwent cystoscopy, those with confirmed bladder tumors underwent either cold cup biopsy or transurethral resection of the bladder tumor. The bladder tumors observed during cystoscopy were matched with the corresponding biopsy results. In cases where a patient had multiple lesions, each lesion’s biopsy result was matched separately.

Tumors were classified into categories based on histopathological results: normal, Ta, T1, T2, and CIS. Additionally, based on the morphological appearance of the tumors observed during cystoscopy, they were categorized as normal, papillary, flat, or mixed lesions. These morphological categories were aligned with pathological classifications for validation process: normal images corresponded to cases without any pathological findings; papillary lesions were primarily associated with Ta cases; flat lesions frequently corresponded to CIS (carcinoma in situ); and mixed lesions reflected characteristics of both papillary and flat lesions, which were often linked to more advanced pathological stages such as Ta, T1, or T2.

The dataset used in this study consisted of 8566 RGB cystoscopic images obtained from 772 patients, with an original resolution of 1080 × 720 pixels, resized to 224 × 224 for classification tasks and 512 × 512 for segmentation tasks, with cropping of unnecessary areas and minor image preprocessing applied, such as neglecting on-image letters and imaging artifacts. Images with low resolution or poor quality, as well as those where lesions were not clearly identifiable due to obstructions like urine sediment or blood clots, were excluded.

Experienced urologists annotated and labeled the lesion areas and the image dataset groups. The dataset was divided into a training set and a validation set with a 4:1 ratio. The dataset exhibited class imbalance, with normal (63.3%) and papillary lesions (24.4%) being predominant, while flat lesions (9.3%) and CIS cases (4.4%) were underrepresented. To address this imbalance, data augmentation techniques such as rotation, zooming, flipping, and shifting were applied to enhance training accuracy and robustness.

While the dataset is currently not publicly available due to institutional regulations and privacy concerns, anonymized access may be considered in the future upon approval by the relevant Institutional Review Board (IRB).

### 2.2. Deep Learning Model

This study involved the development of two deep learning-based models: a bladder cancer classification model, designed to predict pathological results, and a bladder cancer lesion segmentation model, aimed at classifying lesion morphology and delineating lesion margins. The classification model utilized VGG19 with an additional fully connected layer, with sparse categorical cross-entropy defined as the loss function. The segmentation model was created using the Deeplab v3+ model and employed a dice coefficient loss function.

#### 2.2.1. Bladder Cancer Image Classification Model

VGG19 was selected for the bladder cancer classification model because of its proven track record in image classification tasks and its ability to extract deep hierarchical features from images [12]. VGG19 is a deep CNN model recognized for its simple structure and effectiveness in classification of image datasets. The classification model was designed by fine-tuning the pre-trained VGG19 network. The final three layers of the pre-trained network were replaced with a global average pooling layer, a fully connected layer, and a softmax activation layer to classify the images into four categories: normal, papillary, flat lesion, and mixed lesion. The training process utilized the Adam optimizer, setting the learning rate to 0.0001 and the batch size to 32. Data augmentation techniques like random rotation, flipping, and zooming were applied to the training dataset to improve the model’s robustness.

To ensure optimal model performance, the training process involved monitoring validation accuracy and loss to prevent overfitting. Early stopping and learning rate reduction on plateau strategies were employed during training. The dataset was preprocessed to normalize the pixel values and resized to 224 × 224 pixels to match the input requirements of VGG19.

#### 2.2.2. Bladder Cancer Segmentation Model

Deeplab v3+ was chosen for the bladder cancer segmentation model because of its advanced capabilities in semantic segmentation, including capturing multi-scale context with atrous convolution and spatial pyramid pooling [13]. Deeplab v3+ is an advanced architecture for semantic segmentation that enhances performance by incorporating a decoder module. The segmentation model was designed using the Deeplab v3+ architecture with a ResNet-50 backbone. The network was initialized with weights pre-trained on the COCO dataset and fine-tuned on the bladder endoscopic image dataset. The input images were resized to 512 × 512 pixels, and the model training was carried out with the Adam optimizer, employing a learning rate of 0.0001 and a batch size of 16. The dice coefficient loss function was used to address the class imbalance generally present in medical image segmentation tasks. The segmentation model aimed to delineate the boundaries of bladder lesions, classifying pixels into four categories: normal, papillary, flat, and mixed lesions.

Similar to the classification model, the segmentation model utilized data augmentation techniques to enhance generalization. During the training process, model performance was evaluated using monitoring metrics such as the Intersection over Union (IoU) and binary accuracy. To refine the segmentation masks and minimize noise, post-processing steps like morphological operations were applied.

### 2.3. Statistical Analysis

Both models were trained on the bladder endoscopic image dataset, which was divided into an 80:20 ratio for training and validation. The primary evaluation metrics for the classification model were validation accuracy and correlation with biopsy results. The segmentation model was evaluated using the Intersection over Union (IoU) combined with binary accuracy. Data augmentation techniques like rotation, zooming, flipping, and shifting were applied to enhance the models’ robustness and accuracy.

To ensure the reliability and robustness of the results, cross-validation was employed during evaluation. Additionally, the performance of the models was compared against traditional methods to illustrate the advantages of the deep learning approach.

### 2.4. Objective

In the VGG19-based classification, the deep learning model was trained to detect lesion and predict bladder cancer pathology results (Ta low, Ta high, T1, T2, CIS, etc.) using biopsy-confirmed pathologic results annotated images. Validation accuracy and correlation with biopsy results were the primary outcomes, with sparse categorical cross-entropy defined as the loss function.

For Deeplab v3+-based segmentation, the model was trained to segment bladder cancer lesions on cystoscope images. Intersection over Union (IoU) mixed with binary accuracy was used as the evaluation metric, and the dice coefficient was used as the loss function. Visual analysis with the segmentation model was the secondary outcome.

## 3. Results

### 3.1. Baseline Characteristics

**Figure 1:**
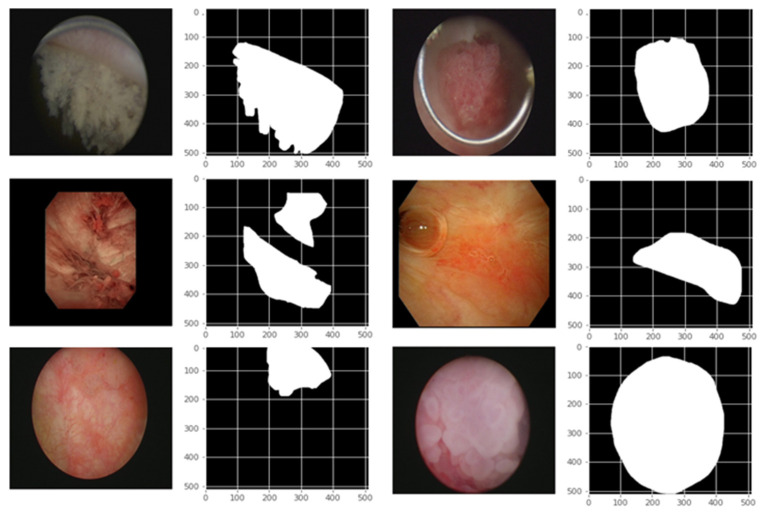
Sample original endoscopy images and segmentation mask of the bladder cancer.

This study utilized data from 772 patients. Each patient’s cystoscope images were annotated with corresponding biopsy results (normal, Ta, T1, T2, and CIS) and morphology (normal, flat, papillary, and mixed). Table 1 presents the patients’ basic characteristics, as well as the distribution of image types and biopsy results. A total of 8,566 images from these 772 patients were used in this study. Experienced urologists labeled the bladder cancer areas and their corresponding pathological results (Figure 1).

**Figure 2:**
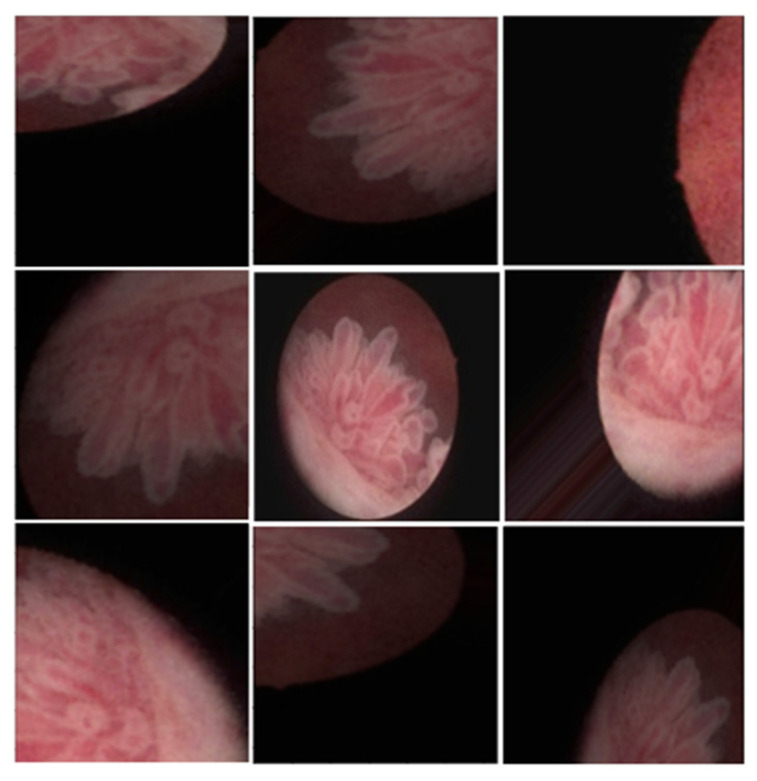
Augmentation of cystoscope images; rotation, zooming, skewness, etc.

The dataset was split into training and validation sets in a 4:1 ratio. Data augmentation techniques, including rotation, zoom in/out, flipping (horizontal/vertical), and height/width shifts, were applied to the images in the dataset (Figure 2).

### 3.2. Development and Validation of VGG19-Based Classification Model

The model using VGG19 was able to detect lesions in the cystoscopic image (accuracy score of 0.912), but classifying them into each biopsy results (normal, Ta, T1, T2, and CIS) did not show meaningful results. The validation loss value and the final loss value, defined as the sparse categorical cross-entropy loss function, were 0.174 and 0.145, respectively.

### 3.3. Development and Validation of a Deeplab v3+-Based Segmentation Model

**Figure 3:**
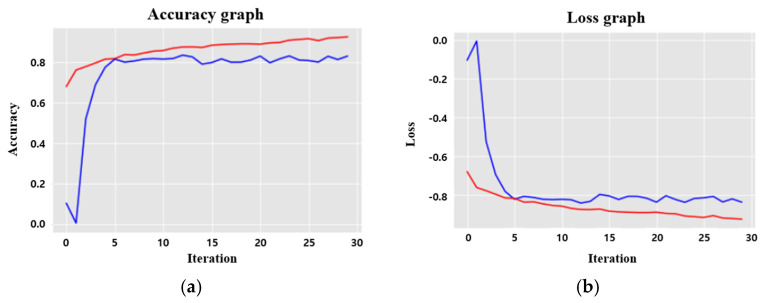
(**a**) Training (red) and validation (blue) loss of segmentation model. (**b**) Training (red) and validation (blue) IoU of segmentation model.

**Figure 4:**
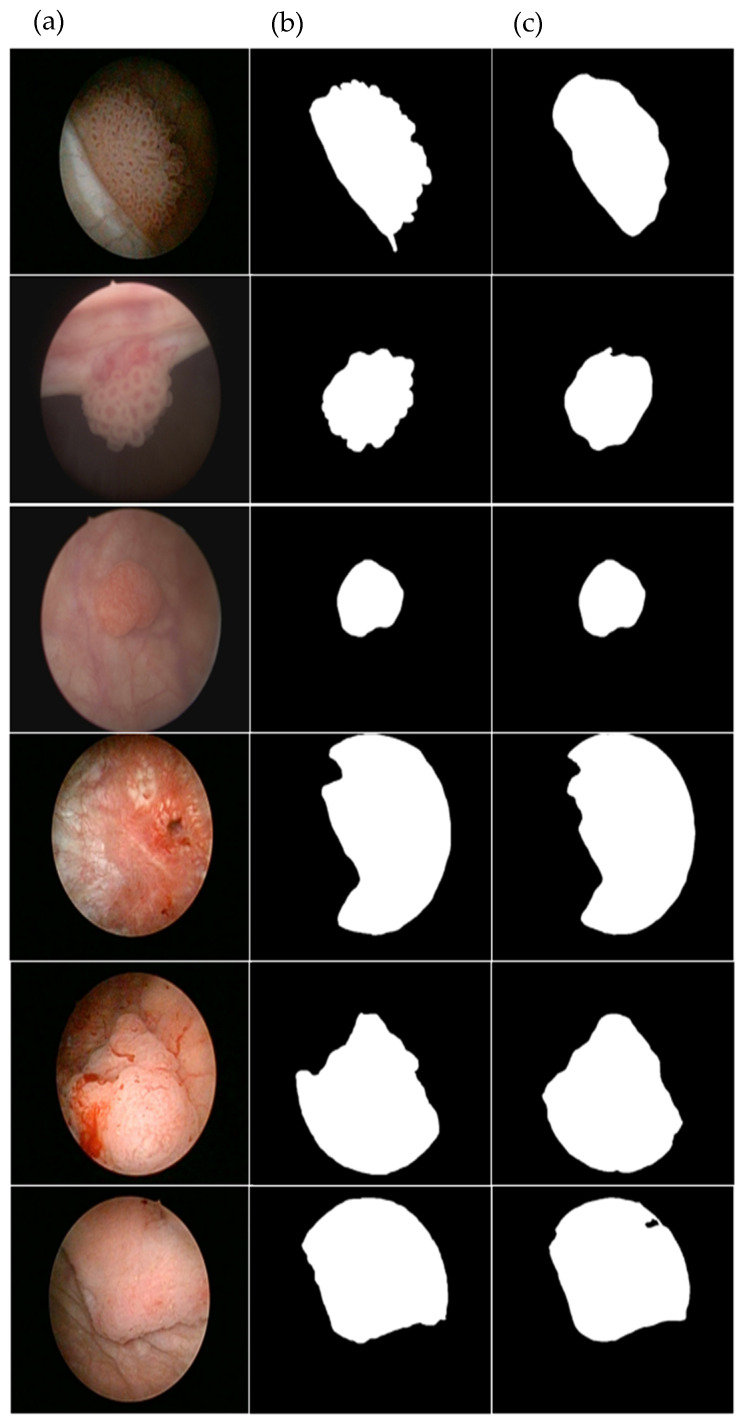
Endoscopic image of bladder cancer and segmentation results. (**a**) Endoscopic image of bladder cancer. (**b**) Image of labeled area by experts. (**c**) Predicted image result of Deeplab v3+.

The segmentation model using Deeplab v3+ achieved an IoU score of 0.833 and a binary accuracy score of 0.951. The validation loss value and final loss value, defined as a dice coefficient loss function, were 0.129 and 0.105, respectively. Mean boundary F1 score (Mean BF) showed 0.931. Figure 3 displays the loss value and IoU of the segmentation model. Additionally, visual analysis was performed, confirming the similarity between the bladder cancer lesion on the cystoscope image recognized by Deeplab v3+ and the lesion labeled by the expert (Figure 4).

## 4. Discussion

Various studies are currently underway to apply deep learning in the field of medicine, with a particular focus on the analysis of medical images obtained from X-rays, CT scans, and MRI scans. Additionally, there are numerous studies exploring the use of deep learning in other areas, such as the analysis of pathological tissue samples, prognosis analysis, and treatment decision-making. In the field of urology, deep learning techniques are being applied to diagnose and treat diseases such as prostate cancer, bladder cancer, and urolithiasis [14,15].

The role of cystoscopy in the diagnosis and treatment of bladder cancer is of utmost importance. However, despite its significance, cystoscopy has its limitations, resulting in a high recurrence rate of bladder cancer. Previous studies have revealed that there is a possibility of missing small lesions or flat lesions such as CIS when using white light cystoscopy [16,17]. Although white light cystoscopy is the standard method for detecting bladder tumors, its diagnostic sensitivity and specificity range from 60% to 70%, and it occasionally overlooks lesions in 10% to 20% of patients. To overcome these limitations, advancements in various technologies, such as narrow band imaging (NBI) or photodynamic diagnosis (PDD), have been developed [18,19]. Meta-analysis has confirmed that using NBI or PDD during tumor resection reduces the recurrence rate and improves diagnostic sensitivity [20]. However, due to the inconvenience associated with both examination methods, white light cystoscopy continues to be the most widely used technique in clinical practice. In addition to advancements in cystoscopy technology, significant research is being conducted in the development of biomarkers for bladder cancer. Manuela et al. [21] found that the expression of let-7c in the urine of bladder cancer patients was up-regulated compared to controls, highlighting its potential as a biomarker for diagnosis or prognosis.

To overcome the limitations of the existing cystoscope and improve diagnostic accuracy, several prior studies have analyzed cystoscope images using deep learning. Ikeda et al. [22] trained a GoogLeNet-based CNN model with 2,102 cystoscopic images, achieving a sensitivity of 89.7% and specificity of 94.0%. Shkolyar et al. [23] developed a CNN-based image analysis platform called CystoNet, demonstrating a sensitivity of 90.9% (95% CI, 90.3–91.6) and specificity of 98.6% (95% CI, 98.5–98.8). Guo et al. [10] utilized the U-Net algorithm for cystoscope image segmentation and achieved an accuracy rate of 98% in detecting bladder cancer lesions using 3500 images from 100 patients. Yoo et al. [24] utilized a mask region-based convolutional neural network with a ResNeXt-101-32x8d-FPN backbone to grade bladder tumors, showing sensitivity of 95.0% and specificity of 93.7%, respectively. These prior studies are summarized in Table 2.

In our study, we employed two deep learning models for the classification and segmentation of cystoscope images. For the classification models using VGG19, we investigated whether the model could predict the pathological results of bladder lesions based on bladder cancer images annotated with biopsy-confirmed pathological results. The model can detect bladder cancer lesion with an accuracy score of 0.912, and the loss value was 0.145. However, the model did not show significant results in distinguishing T1 and T2. This is thought to be due to the lack of sufficient number of T2 lesions required for learning, and the fact that there is no difference in the lesions that are visually visible when T1 and T2 are distinguished by the involvement of the muscle layer. On the other hand, classifying flat lesions as CIS did not show significant results. In this regard, it is believed that it is still a challenge to differentiate flat lesions and further study is needed.

Based on these findings, we believe this approach could be used to predict pathological results and patient prognosis before performing biopsy, thereby improving treatment outcomes. The results are also expected to be useful in the evaluation of chemotherapy response. By applying deep learning in follow-up assessments, it can help determine whether a complete response has been achieved, which can be crucial for avoiding unnecessary treatments. Rocco et al. [25] showed that pelvic lymph node dissection is not needed in T2 or higher bladder cancer patients who have a complete response after neoadjuvant chemotherapy, highlighting the potential benefits of precise response evaluation. Although VGG19 requires substantial memory and computational resources compared to other deep learning models like ResNet or Inception, its simple and regular structure facilitates implementation. Furthermore, VGG19 is well-suited for transfer learning, enabling high performance with limited data in medical fields.

For the segmentation task, the Deeplab v3+ model achieved an IoU of 0.833, a binary accuracy score of 0.951, and a loss value of 0.105. Also, visual analysis showed results similar to those of experts in delineating bladder cancer lesions’ margin. Our study demonstrated meaningful results compared to previous studies using other deep learning models. Deeplab v3+ is particularly advantageous for segmenting bladder cancer lesions during cystoscopy due to its ability to accurately capture and outline complex and variable tumor boundaries. The utilization of Atrous Spatial Pyramid Pooling in Deeplab v3+ effectively integrates multi-scale contextual information, essential for identifying tumors of different sizes and shapes. The encoder–decoder architecture enhances the precision of boundary detection, ensuring that only tumor regions are targeted for removal. Moreover, the flexibility of using pre-trained backbone networks contributes to achieving high accuracy, even when there is variability in endoscopic equipment and imaging conditions across different medical institutions.

While VGG19 and Deeplab v3+ are not the latest models, their simplicity and effectiveness in handling limited datasets justify their selection for this study. Comparative experiments with newer architectures, such as ResNet, EfficientNet, and U-Net variants, revealed marginal improvements in accuracy but reduced specificity and robustness, critical factors in clinical applications. Additionally, the endoscopic images used in this study, unlike conventional grayscale medical images, retain RGB color values and textures that are directly associated with disease characteristics. This unique attribute may have allowed the VGGNet, pre-trained on general image datasets, to leverage these features more effectively, contributing to a slight improvement in accuracy. These findings underscore the importance of balancing model complexity with dataset constraints, a consideration for future studies involving larger, multi-modal datasets.

Our study has several limitations. The relatively small dataset may hinder the model’s ability to generalize effectively. Additionally, this study was conducted using a dataset obtained from a single center, which poses a limitation as the deep learning model was trained on a restricted dataset. However, this study demonstrates the potential of applying deep learning for the early prediction of pathology through the analysis of cystoscope images. Future studies could address this limitation by increasing the size and diversity of the dataset. In cystoscopy, segmenting papillary lesions is relatively easier compared to flat lesions. Therefore, studies aiming to enhance the accuracy of cystoscopy and eliminate false negatives should place greater emphasis on flat lesions. However, our study has a limitation in this regard, as only about 9% of the images used are of flat lesions. Nonetheless, since our model demonstrates high accuracy across all types of lesions, this limitation can be addressed in future follow-up studies. It is worth noting that while VGG19 generally has lower accuracy compared to other CNN-based models like ResNet, it may demonstrate higher accuracy with smaller datasets. Therefore, the high accuracy observed in our classification tasks could be attributed to the limited size of our dataset.

There are areas that can be improved in future research. First, increasing the size of the dataset could enhance accuracy. Additionally, incorporating images that present a limited view due to turbid urine or blood clots, alongside typical lesion images, could provide more challenging scenarios for the deep learning model. Another approach could be to collect datasets from multiple centers, considering differences in endoscopes, light sources, image resolution, and post-processing software used by different hospitals. This diversity could enhance the model’s robustness. Finally, employing CNN architectures that have demonstrated superior performance compared to VGG19 could further improve classification accuracy.

## 5. Conclusions

In this study, we proposed two deep learning models, VGG19 and Deeplab v3+, for the classification and segmentation of bladder lesions using well-annotated datasets of cystoscopic images. Despite the limited dataset size, both models demonstrated high performance in their respective tasks. The VGG19-based classification model achieved an accuracy score of 0.912 in predicting pathological results, while the Deeplab v3+ segmentation model achieved an IoU of 0.833 and a binary accuracy score of 0.951. These results suggest that deep learning models have significant potential in assisting early prediction of bladder cancer pathology and accurate delineation of lesion margins. While our study shows promising results, it has limitations, including the relatively small and single-center dataset. Future research should focus on expanding the dataset size, incorporating images from multiple centers, and exploring more advanced CNN architectures. Despite these limitations, our findings demonstrate the potential of AI-assisted cystoscopy in improving bladder cancer diagnosis and management. With further refinement and validation, these models could become valuable tools in clinical practice, potentially enhancing diagnostic accuracy, standardizing care, and ultimately improving patient outcomes.

## Figures and Tables

**Table 1:** Characteristics of patients and tumor features in this study. Abbreviations: PUNLMP, papillary urothelial neoplasm of low malignant potential; CIS, carcinoma in situ.

	n (%)
Age, median [interquartile range], years	68.2 [61–77.3]
Sex	
Male	590 (76.5)
Female	182 (23.5)
Type of images (Total: 8566)	
Papillary	2086 (24.4)
Flat	794 (9.3)
Mixed	257 (3.0)
Normal	5429 (63.3)
Biopsy results (Total: 573)	
Inverted papilloma	12 (2.1)
PUNLMP	14 (2.4)
Ta, low	102 (17.8)
Ta, high	69 (12.0)
T1, high	93 (16.2)
T2, high	29 (5.1)
CIS	25 (4.4)
Chronic inflammation	22 (3.8)
Urothelial dysplasia	15 (2.6)
Normal	192 (33.5)

**Table 2:** Characteristics and diagnostic accuracy of previous studies using deep learning model for analyzing cystoscope images. (Ac: accuracy, N: normal, Se: sensitivity, Sp: specificity, T: tumor, W: white light image, W/N: white light image and narrow band image).

Reference	Target	Light Source	Number	AI Model	Region of Study	Accuracy
Ikeda et al. [22]	Bladder Cancer	W	2102 images (1671 N, 431 T)	GoogLeNet	Japan	Se: 89.7% Sp: 94.0%
Shkolyar et al. [23]	Bladder Cancer	W	154 patients, 63,480 images	CystoNet	US	Se: 90.9% Sp: 98.6%
Guo et al. [10]	Bladder Cancer	W	100 patients, 3500 images	U-Net	China	Ac: 98%
Yoo et al. [24]	Bladder Cancer	W/N	10,991 images	ResNeXt	Korea	Se: 0.950 Sp: 0.937

## Data Availability

No new data were created or analyzed in this study. Data sharing is not applicable to this article.
